# A Non-Coding RNA Landscape of Bronchial Epitheliums of Lung Cancer Patients

**DOI:** 10.3390/biomedicines8040088

**Published:** 2020-04-13

**Authors:** Yanli Lin, Van Holden, Pushpawallie Dhilipkannah, Janaki Deepak, Nevins W. Todd, Feng Jiang

**Affiliations:** 1 Departments of Pathology, University of Maryland School of Medicine, 10 S. Pine St. Baltimore, MD 21201, USA; linyl1089@163.com (Y.L.); PDhilipkannah@som.umaryland.edu (P.D.); 2 Department of Medicine, University of Maryland School of Medicine, 22 S. Greene St. Baltimore, MD 21201, USA; VHolden@som.umaryland.edu (V.H.); jadeepak@som.umaryland.edu (J.D.); ntodd@som.umaryland.edu (N.W.T.)

**Keywords:** lung cancer, non-coding RNAs, sputum, function, and biomarkers

## Abstract

We propose to systematically identify a non-coding RNA (ncRNA) profile of exfoliated bronchial epitheliums of sputum from lung cancer patients. Bronchial epithelial cells enriched from sputum of 32 lung cancer patients and 33 cancer-free smokers were analyzed by next-generation sequencing to comprehensively characterize the ncRNA profiles. In addition, 108 miRNAs, 88 small nucleolar RNAs, 13 piwi-interacting RNAs, 6 transfer RNAs, 4 ribosomal RNAs, 19 small nuclear RNAs, and 25 long-noncoding (lnc) RNAs displayed a significantly different level in bronchial epitheliums of sputum of lung cancer patients versus cancer-free smokers (all <0.001). PCR analysis confirmed their different expression levels in the sputum specimens. A high expression of SNHG9, an lncRNA, was validated in 78 lung tumor tissues, and the expression was inversely associated with overall survival of lung cancer patients (*p* = 0.002). Knockdown of SNHG9 in cancer cells reduced the cell growth, proliferation, and invasion in vitro and tumorigenesis in vivo. The multiple differentially expressed ncRNAs in bronchial epitheliums may contribute to the development and progression of lung cancer and provide potential biomarkers and therapeutic targets for the disease.

## 1. Introduction

Lung cancer is the number one cancer killer in both men and women [1]. Smoking causes 90% lung cancer cases, of which 85% are non-small cell lung cancers (NSCLC) [1]. NSCLC mainly consists of adenocarcinoma (AC) and squamous cell carcinoma (SCC). Lung tumors develop from a field defect characterized by molecular abnormalities resulted from repeated exposure of the entire airway to the tobacco carcinogens [2]. Airway field changes, such as loss-of-heterozygosity, methylation, and mutation of DNA, and gene expression profiles, were found between primary lung tumors and normal appearing airway epitheliums. Furthermore, prior investigations in field carcinogenesis of the lung airway have proven that the molecular alterations in the large bronchial airways reflected those in primary lung tumors in the distal lung, regardless of the anatomic location relative to the tumors. Therefore, profiling the molecular aberrations in bronchial epitheliums could provide important biologic insights into lung tumorigenesis, and potential biomarkers and therapeutic targets for the disease.

Non-coding RNA molecules (ncRNAs) are a large and diverse class of transcribed RNA molecules and have participated in many biological processes. ncRNAs are divided into two categories based on their length. ncRNAs <200 nucleotides-long are referred to as small ncRNAs, which include transfer (t) RNAs, ribosomal (r) RNAs, micro (mi) RNA, small nucleolar (sno) RNAs, small nuclear (sn) RNAs, piwi-interacting RNA (pi-RNA), etc. [3]. Long noncoding RNAs (ncRNAs) are transcripts longer than 200 nucleotides. ncRNAs play critical roles in the development and progression of cancer [4,5]. For instance, numerous studies including our owns have found that dysregulations of miRNAs can drive multiple processes of lung tumorigenesis by regulating cell cycle, apoptosis, and migration [6,7]. Furthermore, we have demonstrated that aberrant miRNAs detected in sputum can reflect those in primary lung cancer, thus providing potential biomarkers for lung cancer [8,9,10,11,12,13,14].

Aside from miRNAs whose functions in tumorigenesis have been extensively investigated, other ncRNAs (or non-miRNA ncRNAs) are emerging as important players in the development and progression of NSCLC. However, aberrations of other types of ncRNAs in bronchial epitheliums of lung cancer patients remain largely unexplored. Using global next generation sequencing (NGS), in this study, we systematically characterized changes of various types of ncRNAs in exfoliated bronchial epithelial cells enriched from sputum of lung cancer patients. The identified multiple differentially expressed ncRNAs of airway epitheliums may contribute actively to lung cancer development and progression, making them potential selective targets for lung cancer diagnosis and therapeutics.

## 2. Experimental Section

### 2.1. Collecting Sputum and Enriching Exfoliated Bronchial Epithelial Cells

This study was approved by the University of Maryland Baltimore Ethics Committee under ethic codes HP-00040666. Written informed consent was obtained from all enrolled individuals. Before receiving any treatment, participants were instructed to spontaneously cough sputum as described in our previous studies [8,9,10,11,15,16,17,18,19,20,21]. When the participants (mainly former smokers) were not able to spontaneously cough sputum, they underwent sputum induction using a Lung Flute (Medical Acoustics, Buffalo, NY, USA)-based technique as described in our previous work [17]. Sputum was centrifuged at 1000× *g* for 15 min. Cytospin slides were prepared and underwent Papanicolaou staining for evaluating whether the specimens were representative of deep bronchial cells. Sputum has remarkable cell heterogeneity with a large number of macrophages and neutrophils and a limited number of bronchial epithelia [20]. The cell heterogeneity presents a major difficulty in analysis of bronchial epithelial cells of sputum. We have developed a protocol to enrich exfoliated bronchial epithelial cells for analysis of molecular changes in sputum [20]. Using the sample protocol, we purified bronchial epithelial cells from the sputum samples. The bronchial epithelial cell pellet from each sample was washed in phosphate buffered saline (Sigma-Aldrich, St. Louis, MO, USA) and stored at –80 °C until being tested.

### 2.2. NGS Analysis

RNA was extracted from the cells as described in our previous reports [22,23,24,25]. A NanoDrop spectrophotometer (Thermo Fisher Scientific Inc. Waltham, MA, USA) was used to determine the purity and concentration of RNA from OD260/280 readings as described in our published articles [26,27]. Agilent’s RNA 6000 Nano lab-on-a-chip kit and Bioanalyzer (Agilent Technologies) were used to determine the RNA integrity number (RIN) [26,27]. Only RNA with a 260/280 ratio of 1.8–2.0 and a RIN of ≥7 underwent NGS analysis to ensure accurate determination of expression levels of ncRNAs in each sample [26,27]. Illumina HiSeq 2000 system (Illumina, Inc., San Diego, CA, USA) was used to define ncRNA profiles as described in our published articles [26,27]. Briefly, we started with 100 ng RNA, which was diluted in 1 μL nuclease-free dH2O as a ligation reaction. RT and random primers were used to create cDNA. Illumina PE adapters were used to create cDNA libraries. Bioinformatics analysis of the NGS data was performed as previously described [26,27]. In addition, 100 nucleotides of genomic sequences flanking each side of the sequences were extracted, and the RNA secondary structures were predicted using RNAfold. The raw reads were mapped and aligned to sequenced databases (miRbase, piRBase, Genomic tRNA database, Ensembl annotations, Repeat Masker annotation, and the human genes UCSC). The raw reads were also mapped and aligned with lncRNAs by using reference annotation LNCipedia version 3.1. Read counts of identified ncRNAs were normalized to the total number of reads. After normalization, DESeq2 was used to identify ncRNAs that were differentially expressed at 2.0-fold change cutoff in sputum samples of lung cancer patients versus cancer-free smokers.

### 2.3. Reverse Transcription-PCR (RT-PCR) Analysis of NcRNAs in Sputum

To validate the results generated by NGS analysis, we used a different platform, RT-PCR, to test 10 genes in the samples set of specimens. The criteria used the validation was that RT-PCR results of the genes should have ≥2 FC difference between specimens of lung cancer patients compared with controls in the same direction as by NGS, although the scale of changes differed between the two methods. The expression levels of ncRNAs were determined in sputum by using RT-PCR with Taqman assays (Applied Biosystems, Foster City, CA, USA) as previously described [28,29]. The cycle threshold (Ct) was defined as the number of cycles required for the fluorescent signal to cross the threshold. Relative expression of a targeted lncRNA in a given sample was computed using the equation 2−ΔCt, where ΔCt = Ct (targeted lncRNA) – Ct (U6) and U6 was used as an internal control. All experiments were repeated three times, each sample in triplicate. Data represented the mean (±SD) of three independent experiments.

### 2.4. RNA Interference

Specific siRNA targeting RNA sequence of SNHG9 (NCBI accession number NR_003142.2) and corresponding scrambled sequence were designed and synthesized by Integrated DNA Technologies, Inc. (IDT). Transfection was performed by using Opti-MEM medium and Lipofectamine^TM^ RNAiMAX (Invitrogen, California, CA, USA) as previously described [30,31]. All experiments were repeated three times, each sample in triplicate. Data represent the mean (±SD) of three independent experiments.

### 2.5. Cell Viability, Proliferation, and Colony Formation Assays

Cell viability assay was carried out by using Cell Counting Kit 8 (WST-8/CCK8) (Abcam) as previously described [32]. Briefly, cells with a quantity of 5 × 10^3^ cells/well were seeded in a 96-well plate and grown to 80% confluence. CCK-8 reagent was added into each well. Cellular viability was determined by measuring the absorbance of the converted dye at 450 nm at 0, 24, 48, and 72 h. Methylthiazol tetrazolium (MTT) assay was performed to determine cell proliferation as previously described [7,30,31]. Colony Formation Assays were performed as previously described [30,31]. Briefly, the cells were counted and seeded in 6-well plates at a density of 1000 cells per well after transfection. Culture medium was replaced every 3 days and cells allowed to grow for 2, 4, 6, 8, 10, 12, and 14 days. The cells were stained using crystal violet, and each colony with a minimum of 50 cells was counted at each time point. All experiments were repeated three times, each sample in triplicate. Data represented the mean (±SD) of three independent experiments.

### 2.6. Transmigration and Wound Healing Assays

To determine migration and invasion of cells, cells were plated in medium without serum in the top chamber of a transwell (Corning, Moneta, NY, USA). The bottom chamber contained standard medium with 10% FBS. After incubation for 24 h or 48 h, the cells that had migrated to the lower surface of the membrane were fixed with formalin and stained with crystal violet. The migrating cells were examined microscopically and determined by counting the migrating/invasive cells in 5 randomly selected fields using an Olympus BX41 microscope. Photomicrographs were taken using a Qcolor5 digital camera system fitted to this microscope. To determine whether the inhibition of cell migration by SNHG9 knockdown was due to the inhibition of cell proliferation, the cells transfected with siRNA-SNHG9 were treated with aphidicolin (1 mg/mL) (Sigma-Aldrich), a proliferation inhibitor, for 24 h or 48 h. A wound healing assay was performed in 12 well plates (1 × 10^5^ per well). When the cells were grown to 90 to 95% confluences, transfection of cancer cells with siRNA-SNHG9 and scrambled siRNA as well as mock transfection were performed. Wound lines were created manually by scratching the monolayer with a sterile 200 mL pipette tip and migration of the cells was assessed after 24, 48, and 72 h. Pictures were taken using a Nikon inverted phase-contrast microscope (Nikon, Melville, NY, USA). The distance between the parallel lines was measured using ImageJ software. All experiments were carried out at least three times. All experiments were repeated three times, each sample in triplicate. Data represented the mean (±SD) of three independent experiments.

### 2.7. Cell Cycle Analysis by Flow Cytometry

DNA content was analyzed by using flow cytometry as previously described [30,31]. Briefly, harvested cells were washed twice with 5 mM EDTA/PBS and fixed with 95–100% cold ethanol and kept at 4 ℃ overnight. Cells were incubated with RNase A (50 μg/mL), and then stained with Propidium iodide (PI, 10 mg/mL) (Sigma) for DNA content analysis on a FACScan flow cytometer (Becton Dickinson). Statistical analysis of variance (ANOVA) and least significant difference *t*-tests were used to compare cell cycle data of cells with and without specific siRNA treatments. All experiments were repeated three times, each sample in triplicate. Data represented the mean (±SD) of three independent experiments.

### 2.8. Tumorigenicity Assays in Nude Mice

The animal study was performed with the approval of the University of Maryland Baltimore under code IACUC# 0516007. Seven athymic Balb/c, Nu/Nu mice per group were subcutaneously inoculated with 1 × 10^6^ A549 cells transfected with SHGN9-siRNA and 1 × 10^6^ A549 cells with scrambled siRNA, respectively. All the animals were monitored regularly, and tumor growth was measured at regular intervals. The mice were observed for four weeks and then euthanized under deep anesthesia with pentobarbital (Sigma). We calculated volume of the tumors by using formula (length (mm)) × (width (mm)) 2 × 0.52. The tumor size was represented by mean ± SD mm^3^.

### 2.9. Statistical Analysis

Based on our previous studies [26,27,33,34,35,36], the standard deviation of gene expressions was estimated to be 0.757, which was used to guide our power analysis. Since the experiments involved a large number of genes, we controlled the false discovery rate at a 0.025 level. Assuming the proportion of non-expressed ncRNAs was 99%, with sample size of 28 per group, we had 82% power to detect ncRNAs with a fold change (FC) ≥ 2. If the proportion of non-expressed ncRNAs was lower than 99%, the power was even higher with the same sample size [37]. A specific gene was deemed to be significantly differentially expressed if the *p*-value is ≤0.001 with a 2 ≥ FC. Both univariate and multivariate Cox proportional hazard models were applied to assess the effect of clinical variants and SNHG9 expression on survival data of patients. Association of expression of SNHG9 with survival rate was analyzed by using the Kaplan–Meier method. We used ANOVA and least significant difference *t*-tests to compare cell cycle data of the cells with and without specific siRNA treatments. All experiments, except animal study, were repeated three times, each sample in triplicate. For animal study, with seven mice per group, we could have at least 80% statistical power to determine a 2-fold difference in mean tumor volume between groups, tumor volume variation of up to 50% within groups, and a statistical significance level of α = 0.05 [37]. Results of the animal study were analyzed and compared by using Fisher’s exact test between the two groups. A value of *p* < 0.05 was considered statistically significant.

## 3. Results

### 3.1. Study Population

For sputum collection, the participants were recruited at the point of their referral for suspected lung cancer between the ages of 55–80. Exclusion criteria included pregnancy, current pulmonary infection, surgery within six months, radiotherapy within one year, and life expectancy of <1 year. Furthermore, 78 frozen NSCLC tumor tissues and the matched noncancerous lung tissues were obtained from a tissue bank of the University of Maryland Marlene and Stewart Greenebaum Comprehensive Cancer Center. The frozen tissues specimens were collected from consecutive patients with stage I NSCLC who underwent either lobectomy or pneumonectomy from 1999 to 2013. We were able to obtain complete medical records and follow-up data for the patents. Clinical diagnosis of lung cancer was made using histopathologic examinations of specimens obtained by CT-guided transthoracic needle biopsy, transbronchial biopsy, video-assisted thoracoscopic surgery, or surgical resection. The surgical pathologic staging was determined according to the TNM classification of the International Union Against Cancer with the 8th American Joint Committee on Cancer and the International Staging System for Lung Cancer. Histopathological classification was determined according to the World Health Organization classification. Overall survival of the lung cancer patients was obtained by reviewing documentation that the patient was deceased in the medical record or using the Social Security Death Index (SSDI). Demographic and clinical characteristics of the cases and controls from whom sputum were collected are shown in Table 1. Clinical characteristics and histopathological data of the surgically resected tissues specimens are shown in Table 2.

### 3.2. Enriched Bronchial Epithelial Cells of Sputum

Sputum was successfully collected from 65 of 70 subjects, including 32 stage I lung cancer patients and 33 heavy smokers. All the selected sputum specimens were mucoid and of lower respiratory origin as indicated by the presence of bronchial epithelial cells. After being enriched by magnetic-assisted cell sorting, sputum yielded the average yield of bronchial epithelial cells of 386,953 ± 20,425 (mean ± standard deviation, SD). Given that the molecular alterations in bronchial epithelial cells of airway reflect the changes in lung tumors, the analysis of the enriched bronchial epitheliums, rather than mixed sputum cells, for the assessment of the molecular changes of lung tumors could provide an efficient tool for molecular analysis of NSCLC in sputum.

### 3.3. Differentially Expressed ncRNAs in Bronchial Epithelial Cells from Sputum of Stage I NSCLC Patients versus Cancer-Free Smokers

Twenty-eight sputum samples of 32 stage I NSCLC patients and 29 of 33 sputum samples cancer-free smokers (Table 1) were successfully sequenced by using Illumina HiSeq 2500 system (Illumina, San Diego, CA, USA) The rest of the sputum samples had degraded RNA and did not meet our defined minimum requirements, and thus did not proceed with NGS. Each of the enriched bronchial epithelia samples was deep sequenced twice. The number of raw reads obtained per specimen ranged from 17,858,276 to 28,834,167 (average = 23,346,222). Raw reads in the replicates were highly correlated (all *p* < 0.0001), demonstrating that the deep sequencing approach could produce robust results. From the raw reads, an average of 20,896,621 reads (ranging from 15,445,619 to 20,896,621) were filtered. The raw reads were mapped and aligned to the ncRNA sequenced databases. In addition, 2422 miRNAs, 1946 snRNAs, 1522 snoRNAs, 226 piRNAs, 128 tRNAs, 532 rRNAs, and 5061 lncRNAs were annotated in the sputum samples. From the annotated ncRNAs, by using DESeq2, we identified 108 miRNAs, 88 snoRNAs, 13 piRNAs, 6 tRNAs, 4 rRNAs, 19 snRNAs, and 25 lncRNAs, which were differentially expressed at 2.0-fold change cutoff in sputum of cancer patients versus cancer free controls (all *p* ≤ 0.001) (Table S1) (Figure S1). The top 65 altered ncRNAs in bronchial epitheliums of sputum of lung cancer patients versus controls are listed in Table 3. Furthermore, the change levels of the ncRNAs were higher in sputum of lung cancer patients with SCC versus patients with AC (all *p* < 0.05), suggesting that the aberrant ncRNAs presenting in sputum could be more closely related to SCC.

### 3.4. Validation of the ncRNAs in Sputum of Stage I NSCLC Patients versus Cancer-Free Smokers

To confirm the deep sequencing results, we used qRT-PCR to assess expression of four miRNAs, three snoRNAs, and three lncRNAs in the same sputum samples of 28 stage I NSCLC patients and 29 cancer-free smokers (Table 4). One of the 10 ncRNAs was miR-486. Previously published papers from our and others’ laboratories have proven that miR-486 is tumor a suppressor miRNA in NSCLC and is downregulated in specimens of lung cancer patients [6,7,38]. Of the ncRNAs tested, all had >35 Ct value in 90% of the sputum specimens. The results suggested that the ncRNAs were reliably measured in sputum by using a different technique. Furthermore, all the 10 ncRNAs displayed changes by qRT-PCR in the same direction as by NGS, even though the magnitude of changes differed between the two methods (Table 4). Therefore, the aberrant ncRNAs in bronchial epitheliums of lung cancer patients were confirmed by using a different technique. Furthermore, of the 10 tested ncRNAs, there were five genes whose changes were associated with smoking Status (Table S2).

### 3.5. Dysregulation of SNHG9 Contributes to Tumorigenesis of NSCLC

**Figure 1:**
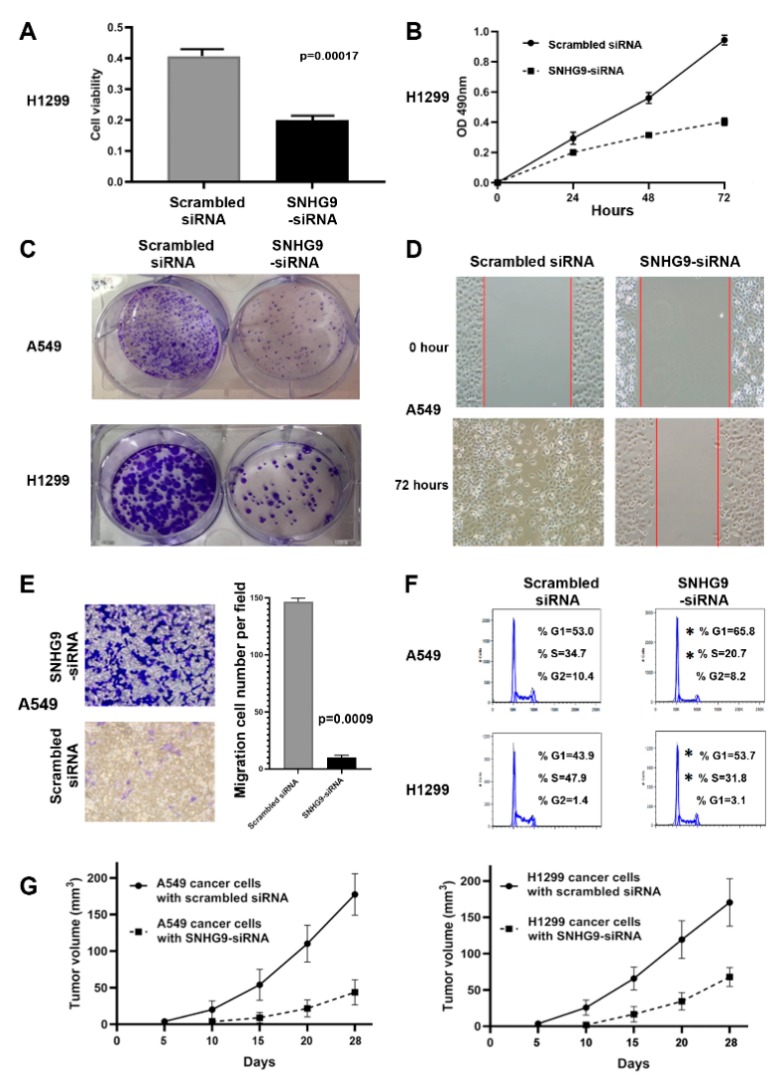
SNHG9 knockdown inhibits the tumorigenicity of A549 and H1299 lung cancer cells. (**A**) SNHG9 knockdown can significantly reduce cell viability by 72 h. The cell viability is determined by a cell viability assay using Cell Counting Kit 8 (Abcam); (**B**) SNHG9 knockdown can suppress cell proliferation in the cancer cells treated with SNHG9-siRNA; (**C**) SNHG9 knockdown significantly inhibits colony formation of cancer cells. The figure shows the results one week after the cells are seeded in the plates (34.8 mm diameter). (**D**) In the wound-healing assays, cancer cells transfected with SNHG9-siRNA show a slower gap closure compared with cells transfected with scrambled siRNA. The figure only shows the results of A549 cells from the time points 0 and 72 h, respectively. (**E**) Transwell migration assays show that SNHG9 knockdown can constrain migration and invasion of A549 cancer cells. The migratory cells are counted, and the results are expressed as the mean number of migratory cells ± SD/selected microscopic field (*n* = 5). The number of migrated cells was evaluated by counting 5 random fields at ×50 magnification. The figure shows the results from the time point 24 h of A549 cells. (**F**) Knockdown of SNHG9 in lung cancer cells (A549 and H1299) elevates the percentage of the cancer cells in the G1/G0 phase and reduces the percentage of S phase (*, *p* < 0.05). (**G**) Quantification of the tumor size over the time in vivo experiment shows that tumor growth in the mice injected with SNHG9-siRNA cancer cells (A549 and H1299) was significantly lower than in the mice injected with the cells transfected with scrambled siRNA. Tumor sizes (mean ± SD^3^) were measured at the indicated intervals and plotted.

Since SNHG9 was one of the lncRNAs that displayed the highest expression level in bronchial epitheliums of sputum from lung cancer patients, we performed in vitro and in vivo analyses to investigate its possible role in tumorigenesis of NSCLC. SNHG9 was highly expressed in ten (76.9%) of the 13 tested cancer cell lines compared with their normal counterpart (Figure S2). Small interfering RNAs specifically targeting SNHG9 (SNHG9-siRNA) were designed that could dramatically reduce SNHG9 expression level in the cancer cells with the treatment (Figure S3). Cell viability was decreased by approximately 47.6% in cancer cells transfected with SNHG9-siRNA compared to cells with scrambled siRNA (*p* = 0.0017) (Figure 1A) (Figure S4A). Furthermore, cell proliferation was reduced in NSCLC cells treated with SNGH9-siRNA compared to cancer cells treated with scrambled siRNA (Figure 1B) (Figure S4B). In addition, suppression of SNHG9 decreased the colony formation, migration, and invasion of the lung cancer cells (Figure 1C–E) (Figure S4C,D). To evaluate whether the inhibition of cell migration of the cancer cells was due to the inhibition of cell proliferation, a proliferation inhibitor, aphidicolin, was added in the Migration assays. Aphidicolin did not affect cell proliferation and migration states of the cancers treated with SNHG9 knockdown (Figures S5 and S6), suggesting that the phenotype was independent of cell proliferation and migration. Moreover, the knockdown of SNHG9 in lung cancer cells increased the percentage of cells in the G1/G0 phase by more than 10%, whereas it reduced the percentage of S phase at least 10%, as compared with cancer cells without knockdown of SNHG9 (all *p* < 0.05) (Figure 1F).

We subcutaneously inoculated A549 or H1299 cancer cells with SNHG9-siRNA, and A549 or H1299 cancer cells with scrambled siRNA into flanks of nude mice. Tumors were visible as early as five days in the mice that received scrambled siRNA-cancer cells. On 14 days post-injection, tumors appeared in all the seven mice injected with the scrambled siRNA-cancer cells. However, on 14 days post-injection, only five tumors appeared in the seven mice injected with SNHG9-siRNA-cancer cells. On 17 days post-injection, tumor appeared in another two mice injected with SNHG9-siRNA-cancer cells. Overall, tumor growth in the mice injected with SNHG9-siRNA cancer cells was significantly lower than in the mice injected with the cells transfected with scrambled siRNA (Figure 1G). The tumors generated from scrambled siRNA-cancer cells were significantly larger compared to those produced from the cancer cells with SNHG9-siRNA at the end of observation (28 days) (174.72 ± 96.69 mm^3^ vs. 24.78 ± 12.98 mm^3^, *p* = 0.03). These in vivo findings in ectopic xenograft mouse models are consistent with the in vitro observations, and hence support that suppression of SNHG9 could inhibit in vivo tumorigenicity of lung cancer cells.

### 3.6. Upregulation of SNHG9 Is Associated with Advanced Stage and Survival of Lung Cancer Patients

**Figure 2:**
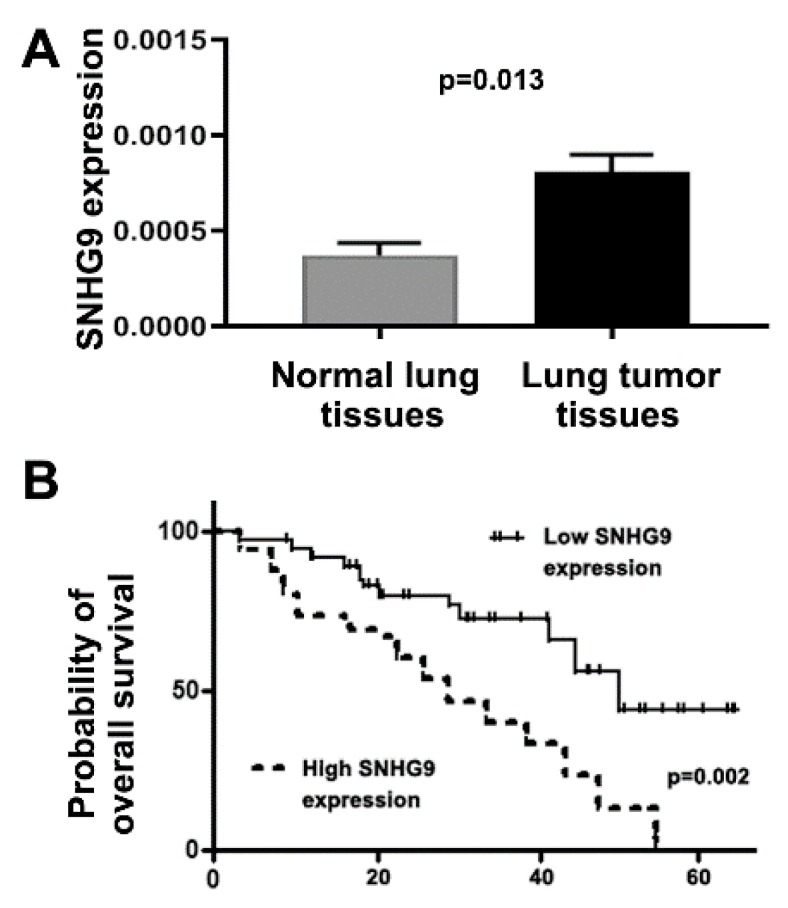
Expression of SNHG9 determined by using RT-PCR in surgically resected lung tumor tissues and the matched noncancerous lung tissues of 78 lung cancer patients. (**A**) SNHG9 has a higher expression level in lung tumor tissues compared with the corresponding noncancerous lung specimens (*p* = 0.013). (**B**) The 78 NSCLC patients were classified into two groups according to a median SNHG9 expression value in the lung tumor tissues (0.00034 ± 0.0001). The patients with high expression of SNHG9 in the lung tumor tissues had significantly shorter survival time than those with low expression level of the gene (*p* = 0.001).

To investigate clinical significance of SNHG9 dysregulation, we used qRT-PCR to determine expression of the gene in frozen surgically resected lung tumor tissues and matched noncancerous lung tissues of 78 patients with NSCLC (Table 2). U6 was used as an internal control gene to determine relative expression of SNHG9 in the tissue specimens. SNHG9 displayed a significantly higher expression level in lung tumor tissues compared with the corresponding noncancerous lung specimens (=0.013) (Figure 2A). Both univariate and multivariate analyses showed that expression of the SNHG9, age, and stage were significantly associated with survival of the patients (all *p* < 0.05) (Tables S3 and S4). No statistically significant correlation was observed between SNHG9 expression in the tissue specimens and patient age, sex, and tumor histological type (all *p* < 0.05), except smoking history of the patients (*p* < 0.05). SNHG9 expression was positively associated with stage of lung cancer (*p* < 0.05). The 78 NSCLC patients were classified into two groups according to a median SNHG9 expression value (0.00034 ± 0.0001) in lung tumor tissues. A high SNHG9 expression in tumor tissues was a predictive of shorter overall survival time (*p* < 0.01). In addition, the Kaplan–Meier Curve indicated that the lung cancer patients with a high SNHG9 expression level had a poor survival compared with the patients who had a lower level of the SNHG9 in the tumor tissues (*p* = 0.001) (Figure 2B). Therefore, the assessment of SNHG9 might provide a potential approach for predicting outcome of NSCLC.

## 4. Discussion

In this study, we identified a broad spectrum of ncRNA features in bronchial epitheliums of sputum from NSCLC patients, which include seven major types of ncRNAs. Of the various types of ncRNAs, miRNAs have been extensively investigated for the functions in lung cancer development and progression. For instance, analyze epithelial brushings from the main stem-bronchus, Pavel et al. identified 42 miRNAs that showed abnormal expressions in lung cancer patients [39]. However, collecting bronchial epithelial cells from the mainstem bronchus through bronchoscopy is an invasive procedure. Furthermore, using NGS to analyze surgically resected lung tumor tissues, we identified miRNA and snoRNA changes in the tissue specimens of lung cancer patients [26,27,40]. This present study is the first to systematically and comprehensively characterize changes of ncRNAs, including miRNAs, in exfoliated bronchial epithelial cells enriched from sputum of lung cancer patients by using global NGS. The differentially expressed miRNAs in bronchial epitheliums enriched from sputum could contribute to the development of lung tumor.

Besides these miRNAs, other ncRNAs (non-miRNA ncRNAs), including 23 snRNAs, 93 snoRNAs, 13 piRNAs, 7 tRNAs, 15 rRNAs, and 25 lncRNAs, also differentially expressed in bronchial epitheliums of lung cancer patients compared with those of cancer-free smokers. The non-miRNA ncRNAs are gaining prominence and more actively involved in carcinogenesis than previously though. For example, snoRNAs serve as guides for the 2′-*O*-ribose methylation of rRNAs or snRNAs and their isomerization of uridine residues into pseudo uridine [41]. Our previous studies have shown that snoRA42 has oncogenic function in the development and progression of NSCLC by regulating features of lung tumor-initiating cells [30,31]. Such pleiotropy of snoRA42 dysregulation could be achieved partially through increased apoptosis of NSCLC cells in a p53-dependent manner [30]. piRNAs are Dicer-independent and can interact with the PIWI subfamily of Argonaute proteins involved in the regulation of genome stability. A high expression level of piR-651 in lung tumor tissues is associated with cancer progression in the patients with NSCLC [42]. Upregulation of piR-651 could induce NSCLC progression via the cyclin D1 and CDK4 pathway [42,43,44]. However, inhibition of piR-651 reduced cell proliferation and significantly increased the apoptotic rate of lung cancer cells [44]. rRNA has essential functions for protein synthesis in all living organisms [45]. Recent studies have shown that rRNA expression and modifications have an important function in cancer progression. Increased rRNA expression was associated with cancer development in prostate and cervical cancer [46]. In colorectal cancer, high expression of the pre-45S rRNA promoted G1/S cell-cycle transition and was associated with poor prognosis [47]. tRNAs have the function of transporting the amino acids to the ribosome. Some tRNAs and tRNA derivatives have been suggested to involve in proliferation, metastasis, and invasiveness of cancer cells [48]. An elevated expression level of tRNAs-Leu and tRNAs-Val was found in lung tumor tissues [49]. Methionyl-tRNA synthetase overexpression was associated with poor clinical outcomes in lung cancer [50]. snRNAs have profound effects on the cellular transcriptome and are complexed with small nuclear ribonucleoproteins [51]. Furthermore, snRNAs could recognize 5′ and 3′ intron/exon boundaries during splicing of introns from pre-messenger RNA transcripts. snRNAs might have important functions in cancer development and progression, as well as in drug resistance [3]. For instance, U1 snRNP could regulate cancer cell migration and invasion in vitro [52]. Nonetheless, further investigation of the multiple ncRNAs whose changes exist in bronchial epitheliums would be important to understand the mechanism of field cancerization in lung carcinogenesis.

lncRNAs become one of the focuses in the research field, since they play a central role in numerous physiological and pathological processes of a wide variety of diseases. Particularly, lncRNAs can regulate different molecular signaling pathways via changing gene expression, and, therefore, are implicated in numerous mechanisms of lung carcinogenesis. We have found that expression levels of SNHG1 and RMRP are reliably measured in plasma, and the lncRNAs may provide cell-free circulating biomarkers for lung cancer [28]. Interestingly, our identified altered lncRNAs in bronchial epitheliums of lung cancer patients include the previously characterized lung cancer-associated lncRNAs. Dysregulation of the lncRNAs in bronchial epitheliums may contribute to the field cancerization in lung carcinogenesis. The SNHG family includes numerous members, whose dysregulations are associated with a variety of malignancies [53]. Each SNHG might contribute to tumorigenesis via multiple molecular regulatory mechanisms [53]. For example, some SNHGs could act as sponges of microRNAs to inhibit the roles of miRNAs. They can also bind proteins to influence target genes or impact tumorigenesis via different signaling pathways, including the EMT, Wnt, PIK3CA, NF-κB, and TP53 signaling pathways [54]. Herein, we found that SNHG9 may have an important function in pathogenesis of NSCLC, since its transient knockdown contributes to NSCLC cell growth, proliferation, and invasion. In addition, the restriction of cancer cell growth and proliferation by the SNHG9 downregulation were accompanied by an accumulation of cells in the G1 phase and a decreased rate of S-phase. Moreover, the in vitro tumorigenicity of SNHG9 was confirmed by the observation in ectopic xenograft mouse models. The elevated SNHG9 expression was frequently observed in lung tumor tissues. Importantly, SNHG9 expression in tumor tissues is positively related to advanced stages of lung cancer and inversely correlated with survival of the patients. The results obtained from the clinical specimens provide further evidence that the dysregulation of SNHG9 contributes to lung cancer development and progression. The detection of SNHG9 aberrations might be a useful approach to identify NSCLC patients who have poor prognoses. Furthermore, the in vitro and in vivo data of siRNA-mediated silencing of the lncRNA would form the basis developing novel therapeutic targets for the malignancy. However, the molecular mechanisms underlying the biologic effects of SNHG9 in lung tumorigenesis remain unknown. We are performing a new study to investigate possible mechanisms or pathways, by which SNHG9 might participate in the development and progression of NSCLC. Furthermore, of the ncRNAs that displayed the highest expression level in bronchial epitheliums of sputum from lung cancer patients, only SNHG9 was further analyzed by in vitro and in vivo approaches in this present study. Our ongoing studies are investigating possible roles of other differentially expressed ncRNAs in tumorigenesis of lung cancer.

Since the molecular abnormalities in the large bronchial airways reflect those in primary lung tumors in the distal lung, molecular changes, such as aberrant ncRNAs in epithelial cells collected from the normal-appearing mainstem bronchus of smokers could be developed as biomarkers for NSCLC. As a mirror to lung diseases, sputum contains bronchial epithelial cells from the lungs and lower respiratory tract. Examination of the exfoliated bronchial epitheliums of airway in sputum might detect the lung tumor-related molecular alterations, and hence provide a non-invasive and specific means for diagnosis of NSCLC. We have demonstrated that, due to their relative resistance to nucleases, miRNAs are highly stable in sputum, providing biomarkers for lung cancer [8,9,11,12,22,23]. Given that the various types of abnormal ncRNAs in bronchial epitheliums that have highly different and active roles in lung tumorigenesis via numerous cellular pathways, developing the non-miRNA ncRNAs as sputum biomarkers and integrating them with the existing miRNAs biomarkers might have a synergistic effect for NSCLC detection.

## 5. Conclusions

By transcriptomic sequencing airway epithelial cells of sputum, we identified multiple differentially expressed ncRNAs in bronchial epitheliums of lung cancer patients. Further functional studies of the broad spectrum of ncRNA features would deepen our understanding of the molecular mechanisms driving lung carcinogenesis and provide potential biomarkers and therapeutic targets for the disease.

## Figures and Tables

**Table 1:** Demographic and clinical data of 32 lung cancer patients and 33 cancer-free smokers.

	Lung Cancer Patients	%	Cancer-Free Controls	%
Parameter				
Mean of age (years)	68.2		65.6	
Gender				
Men	21	65.6	22	66.7
Women	11	34.4	11	33.3
Race				
White American	23	71.9	23	69.7
African American	9	28.1	10	30.3
Mean of smoking pack-years	49.3		37.8	
Stage	All are stage I NSCLC			
Histology				
Adenocarcinoma	16	50		
Squamous cell carcinoma	16	50		

Abbreviations: NSCLC, non-small cell lung cancer.

**Table 2:** Demographic and clinical characteristics of 78 NSCLC patients.

Characteristics	Number of Patients in Each Category (%)
Mean of age (years) at diagnosis	66.9
Sex	
Male	52 (66.7)
Female	26 (33.3)
Race	
White	49 (62.8)
Black	29 (37.2)
Smoker	
Yes	70 (89.7)
No	8 (10.3)
Tumor histology	
Squamous cell carcinoma	39 (50.0)
Adenocarcinoma	39 (50.0)
T stage	
I	30 (38.5)
II	29 (37.2)
III–IV	19 (24.3)

NSCLC, non-small cell lung cancer.

**Table 3:** Fold-change (FC) of top 65 altered ncRNAs in bronchial epitheliums of sputum of lung cancer patients versus controls *.

Genes	FC	Genes	FC
miRs		piRNAs	
MIR-9-1	28.146	piR-004987	5.637
MIR-577	21.745	piR-020809	5.058
MIR-410	21.007	piR-023338	−3.489
MIR-487B	18.242	piR-011186	−4.161
MIR-409	14.424	tRNAs	
MIR-338	−7.723	TRNAV33P	16.688
MIR-486	−8.086	TRNAE27P	15.772
MIR-135A1	−19.011	TRNAG34P	2.159
MIR-184	−28.380	TRNAG32P	16.774
snRNAs		TRNAE40P	2.107
RNU5E-1	32.515	TRNAK42P	−2.006
U4	11.005	rRNAs	
RNU7-1	6.874	RN5-8S5	4.936
RNU4ATAC	6.400	RN5-8S3	2.008
RNU5A-1	5.736	RN5-8S2	5.151
snoRNAs		RN5248	−2.188
SNORD114-20	43.008	lncRNAs	
SNORD113-5	36.658	SNHG9	8.474
SNORD114-25	31.656	SNHG2	7.568
SNORD114-28	30.173	MEG8	7.078
SNORD114-26	22.244	LINC00461	7.786
SNORD113-7	19.579	SNHG11	6.635
SNORD114-21	17.826	GAS5	−3.082
SNORD33	16.235	TUG1	−4.866
SNORD114-23	16.090	PANDAR	−5.099

* All *p* > 0.05.

**Table 4:** Expression of 10 ncRNAs detected by NGS and RT-PCR in sputum of stage I non-small cell lung cancer patients vs. cancer-free smokers.

**Expression Levels of the ncRNAs Detected by NGS**				
**ncRNAs**	**Median of RPM in NSCLC Patients**	**Median of RPM in Controls**	**Log2 Fold Change (FC) of Patients/Controls**	***p*-Value**
miRNAs				
miR-21	39789.25	578.96	6.10	<0.001
miR-31	4800.34	973.20	2.30	<0.001
miR-210	5935.71	226.72	4.71	<0.001
MIR-486	140.87	37632.26	–8.06	
snoRNAs				
snoRD66	4822.28	151.60	4.99	<0.001
snoRD78	3983.48	161.80	4.62	<0.001
snoRA42	4518.27	86.72	5.70	<0.001
lncRNAs				
SNHG9	70896.26	198.99	8.48	<0.001
H19	6073.51	160.89	5.24	<0.001
HOTAIR	2616.69	309.18	3.08	0.0060
**Expression levels of the ncRNAs detected by RT-PCR**				
**ncRNAs**	**Mean of Level in NSCLC Patients**	**Mean of Level in Controls**		***p*-Value**
miRNAs				
miR-21	297.26	7.16	5.38	<0.001
miR-31	4.89	0.75	2.70	<0.001
miR-210	178.94	12.86	3.80	<0.001
MIR-486	19	378	−4.31	
snoRNAs				
snoRD66	1.71	0.04	5.42	<0.001
snoRD78	1.86	0.12	3.95	0.001
snoRA42	2.65	0.1	4.73	<0.001
lncRNAs				
SNHG9	16.78	0.33	5.67	<0.001
H19	9.34	0.86	3.44	<0.001
HOTAIR	3.11	0.38	3.03	<0.001

Abbreviations: NSCLC, non-small cell lung cancers; NGS, next generation sequencing; RT-PCR, reverse transcriptase PCR; RPM, reads per million.

## Supplementary Materials

Supplementary materials can be found at https://www.mdpi.com/2227-9059/8/4/88/s1.
