# Supplementary material for: A Non-Coding RNA Landscape of Bronchial Epitheliums of Lung Cancer Patients
Source: Biomedicines. 2020 Apr 13;8(4):88. doi: 10.3390/biomedicines8040088 (PMC7235744; doi:10.3390/biomedicines8040088)
Supplement: Supplementary file 1 [file biomedicines-08-00088-s001.pdf]

Supplementary Materials: A Non-Coding RNA Landscape of Bronchial Epitheliums of Lung Cancer Patients

Supplementary Table S1. Fold-change (FC) of various types of ncRNAs in bronchial epitheliums of sputum of lung cancer patients versus cancer-free smokers.

Genes	FC	Genes	FC	Genes	FC	Genes	FC	Genes	FC
**miRs**		MIR-31	2.3380	**snRNAs**		SNORD1C	5.9102	**piRNAs**	
MIR-9-1	28.1462	MIR-345	2.2716	RNU5E-1	32.5154	SNORD121A	5.8408	piR-004987	5.6373
MIR-9-2	28.1361	MIR-214	2.2587	U4	11.0046	SNORD18B	5.8326	piR-020809	5.0580
MIR-9-3	28.1327	MIR-2355	2.2538	RNU7-1	6.8739	SNORD112	5.8037	piR-016240	4.0580
MIR-577	21.7445	MIR-150	2.2456	RNU4ATAC	6.3999	SNORA42	5.6923	piR-016946	4.0580
MIR-410	21.0071	MIR-449B	2.2072	RNU5A-1	5.7364	SNORD80	5.6077	piR-021190	3.0580
MIR-487B	18.2420	MIR-944	2.2022	RNU4-2	4.2876	SNORD11	5.6059	piR-004987	2.9466
MIR-409	14.4240	MIR-92A1	2.1177	U2	3.8470	SNORA45	5.3059	piR-004987	2.3808
MIR-194-2	13.6073	MIR-29A	2.0500	RNU2-5P	3.1170	SNORD18C	5.0454	piR-007109	-2.8849
MIR-539	13.1634	MIR-653	2.0446	RNU5B-1	2.9721	SNORA34	4.9745	piR-000520	-2.8849
MIR-194-1	13.0849	MIR-92A2	2.0236	RNU2-2	2.7230	SNORD78	4.6590	piR-011547	-2.3885
MIR-369	12.4291	MIR-625	2.0078	RNU1-1	2.7109	snR39B	4.4313	piR-023338	-2.7161
MIR-432	10.8032	MIR-130A	-2.0000	RNU1-4	2.7084	SNORD35A	4.3511	piR-023338	-3.4893
MIR-301B	10.7921	MIR-181A1	-2.0272	RNU1-3	2.7084	SNORD45C	4.3247	piR-011186	-4.1610
MIR-192	9.8875	MIR-181A2	-2.1028	RNU1-2	2.7084	SNORD88B	4.3048	**tRNAs**	
MIR-215	9.6398	MIR-138-2	-2.1105	RNU2-4P	2.5710	SNORD113-3	4.2199	TRNAV33P	16.6884
MIR-370	9.5159	MIR-203	-2.1121	RNU11	2.3087	SNORD75	4.2081	TRNAK42P	-2.0064
MIR-376C	9.1398	MIR-190B	-2.1238	RNU2-6P	2.0258	SNORD37	4.0793	TRNAG34P	2.1591
MIR-654	9.0507	MIR-138-1	-2.1473	RNU4-1	2.0085	SNORD38B	4.0601	TRNAG32P	16.7736
MIR-889	8.6332	MIR-509-2	-2.1793	RNU8	-2.0098	SNORD10	4.0269	TRNAE40P	2.1071
MIR-493	8.3036	MIR-509-1	-2.1793	**snoRNAs**		SNORA8	4.0171	TRNAE27P	15.7722
MIR-224	7.5661	MIR-133A1	-2.1805	SNORD114-20	43.0084	SNORA64	4.0011	**rRNAs**	
MIR-377	6.8836	MIR-133A2	-2.1805	SNORD113-5	36.6577	SNORD27	3.9490	RN5S248	-2.1877
MIR-877	6.3188	MIR-101-1	-2.1821	SNORD114-25	31.6557	SNORD49B	3.8630	RN5-8S5	4.9357
MIR-136	6.2961	MIR-100	-2.1883	SNORD114-28	30.1733	SNORD36A	3.8035	RN5-8S3	2.0076
MIR-381	6.1287	MIR-101-2	-2.1911	SNORD114-26	22.2437	SNORD3D	3.7433	RN5-8S2	5.1505
MIR-21	6.0924	MIR-146A	-2.1853	SNORD113-7	19.5793	SNORD124	3.6942	**lncRNAs**	

MIR-127	5.7509	MIR-143	-2.2673	SNORD114-21	17.8260	SNORD103B	3.5551	SNHG9	8.4740
MIR-382	5.2319	MIR-10B	-2.3873	SNORD33	16.2345	SNORD103A	3.5551	SNHG2	7.5675
MIR-210	4.7710	MIR-99A	-2.3292	SNORD114-23	16.0901	SNORD63	3.5443	MEG8	7.0784
MIR-299	4.7698	MIR-509-3	-2.3578	SNORD113-6	14.9458	SNORD4B	3.5196	LINC00461	7.7856
MIR-193B	4.4713	MIR-362	-2.3649	SNORD19B	14.3585	SNORD79	3.4508	SNHG11	6.6354
MIR-134	4.2657	MIR-373	-2.5322	SNORD66	4.9992	SNORD58C	3.4494	CAR10	6.2654
MIR-135B	4.2297	MIR-218-1	-2.5753	SNORD114-9	12.8312	SNORD47	3.3880	H19	5.2456
MIR-130B	4.0313	MIR-218-2	-2.6421	SNORD113-9	12.5820	SNORD3B-2	3.3793	DLX6-AS1	4.6785
MIR-708	3.8715	MIR-374B	-2.7736	SNORD114-15	11.7975	SNORD3B-1	3.3793	MALAT1	4.1775
MIR-337	3.6670	MIR-126	-2.7777	SNORD28	11.0206	SNORA57	3.3735	RGMBAS1	3.8646
MIR-200C	3.2296	MIR-598	-2.7914	SNORA68	10.9916	SNORD91B	3.3734	PVT1	3.8635
MIR-411	3.1703	MIR-139	-2.8565	SNORA16A	8.8388	SNORD121B	3.3329	BCYRN1	3.5634
MIR-1307	3.0606	MIR-223	-3.1722	SNORD11B	8.6210	SNORD96A	3.3315	HOTAIR	3.0945
MIR-3607	3.0477	MIR-146B	-3.2749	SNORD114-3	8.1622	SNORD73A	3.2209	RMRP	3.0001
MIR-183	3.0473	MIR-551B	-3.4310	SNORD113-8	7.9298	SNORD105B	3.2203	SOX2-OT	2.9756
MIR-182	3.0432	MIR-34C	-4.3615	SNORA18	7.6745	SNORD19B	3.2120	HNF1A-AS1	2.9356
MIR-200B	2.9627	MIR-1-1	-4.6336	SNORD54	7.6737	SNORD5	3.2026	CCAT2	2.8367
MIR-205	2.7952	MIR-1-2	-4.6650	SNORD113	7.6060	SNORD88A	3.1115	LUADT1	2.5766
MIR-429	2.6851	miR-1979	-4.8376	SNORA28	7.5867	SNORD25	3.1090	ZXF1	2.5523
MIR-96	2.6588	MIR-34B	-4.8821	SNORD114-12	7.5184	SNORA3	3.0994	ANRIL	2.3036
MIR-199A1	2.6160	MIR-144	-5.1747	SNORD77	7.2402	SNORD114-17	3.0897	MEG3	-2.8655
MIR-199A2	2.6098	MIR-451A	-5.3845	SNORD46	7.1582	snoU13	3.0726	SPRY4-IT1	-2.5675
MIR-301A	2.5580	MIR-30A	-6.7271	SNORD16	7.1378	SNORD4A	3.0527	GAS5	-3.0820
MIR-199B	2.5492	MIR-338	-7.7232	SNORA71D	6.8841	SNORD14D	3.0420	TUG1	-4.8657
MIR-549	2.5312	MIR-486p5p	-8.0625	SNORD46	6.7108	SNORD60	3.0107	PANDAR	-5.0987
MIR-452	2.4256	MIR-135A1	-19.0114	SNORD114-1	6.5720	SNORD115	-2.0096		
MIR-375	2.4061	MIR-184	-28.3800	SNORA75	6.2625	SNORD89	-2.5482		
MIR-320C2	2.3995			SNORD72	6.1121				

Supplementary Table 2. Pearson's correlation coefficient test shows that the ncRNAs are relative to smoking status.

ncRNAs	Pearson's coefficient	p-value
MIR-21	-0.208	0.0068
MIR-210	-0.195	0.0113
miR-486	-0.244	0.0014
snoRA42	-0.162	0.04
SNHG9	-0.173	0.0251

Supplementary Table S3. Univariate Cox Proportional Hazards regression analysis of covariates in relation to survival of patients.

**Covariate**	**Overall survival**
Age	0.028
Sex	0.645
Smoking Status	0.295
Tumor histology	0.472
Stage	0.029
SNHG9 overexpression	0.002

The numbers in the table represent P values calculated with the Wald test. P values <0.05 were considered statistically significant.

Supplementary Table S4. Multivariate Cox proportional hazards regression analysis to evaluate the prognostic value of snoRNA signature and clinical parameters.

**Covariate**	**Overall survival**
Age	0.037
Sex	0.521
Smoking Status	0.469
Tumor histology	0.482
Stage	0.028
SNHG9 overexpression	0.001

Numbers in the table represent P values calculated with the Wald test.

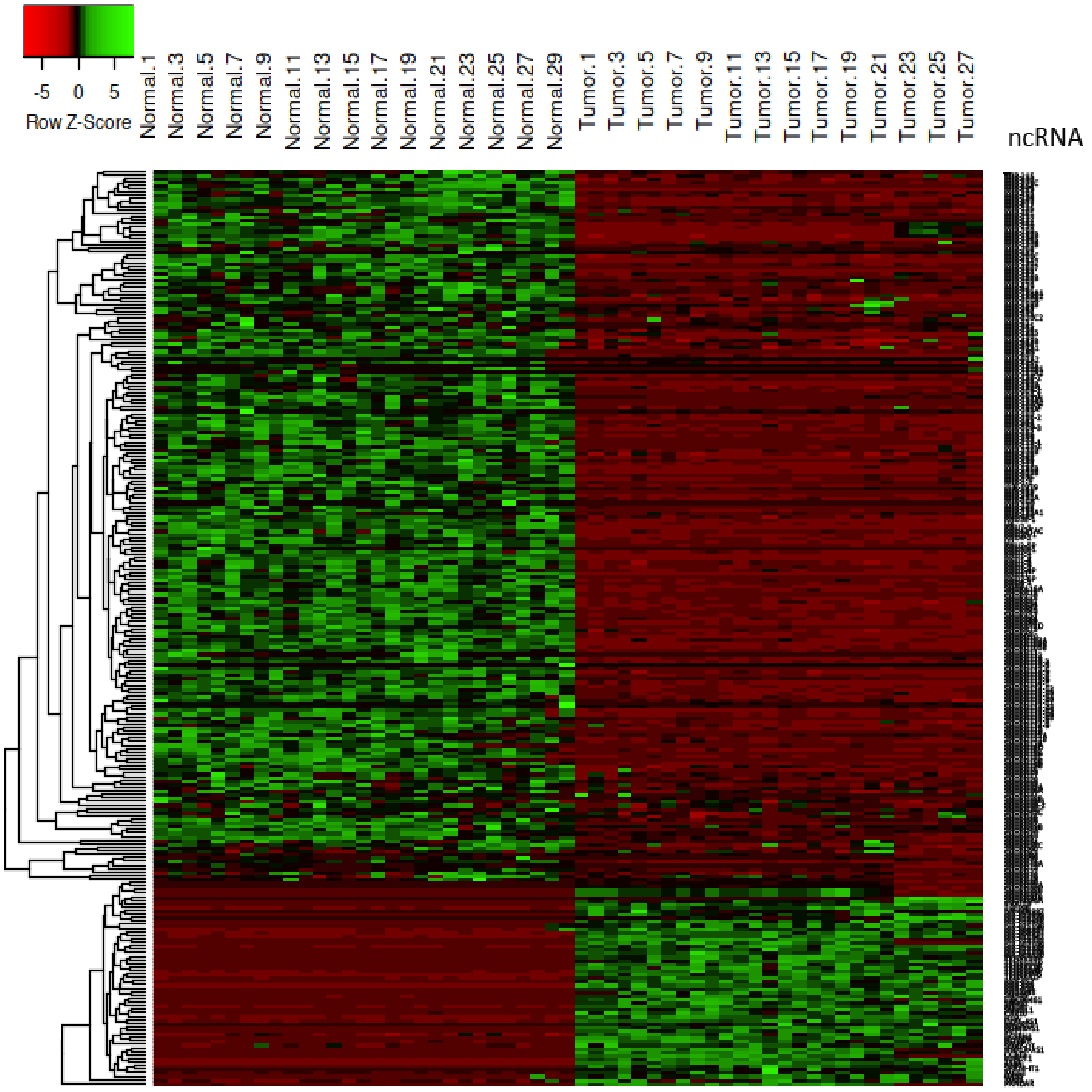

Supplementary Fig 1. Heatmap of individual sputum samples of 29 cancer-free smokers and 28 lung cancer patients. The ncRNA expression levels exhibited ≥2.0 fold changes and $p \leq 0.05$ are presented. Each column represents an individual sample and each row represents a single ncRNA. Expression level of each ncRNA in a single sample is depicted according to the color scale.

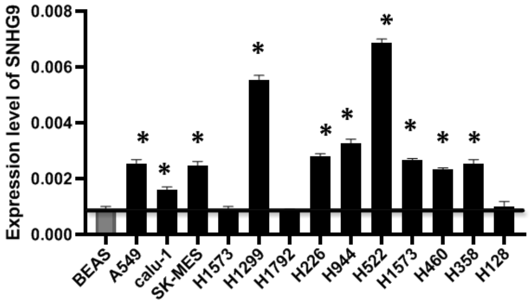

<u>**Supplementary Fig 2.** Expression level of SNHG9 in a normal lung cell line and 13 lung cancer cell lines. U6 was used as an internal control gene to normalize RT-PCR data to determine relative expression of SNHG9 in the cell lines. Of the 13 lung cancer lines, 10 had a higher expression level (P<0.05) compared with the normal lung cell line (BEAS). *, p<0.05.</u>

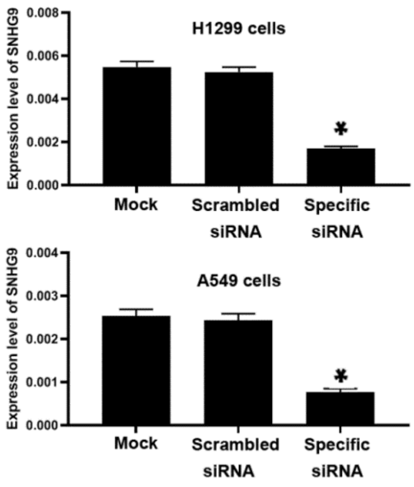

<u>**Supplementary Fig 3.** SNHG9-siRNA can reduce SNHG9 expression in cancer cells. SNHG9 was efficiently and specifically reduced by SNHG9-siRNA in both H1299 and A549 lung cancer cells. The figure shows expression levels of SNHG9 in the cancer cells 48 hours after the transfection (*p<0.001).</u>

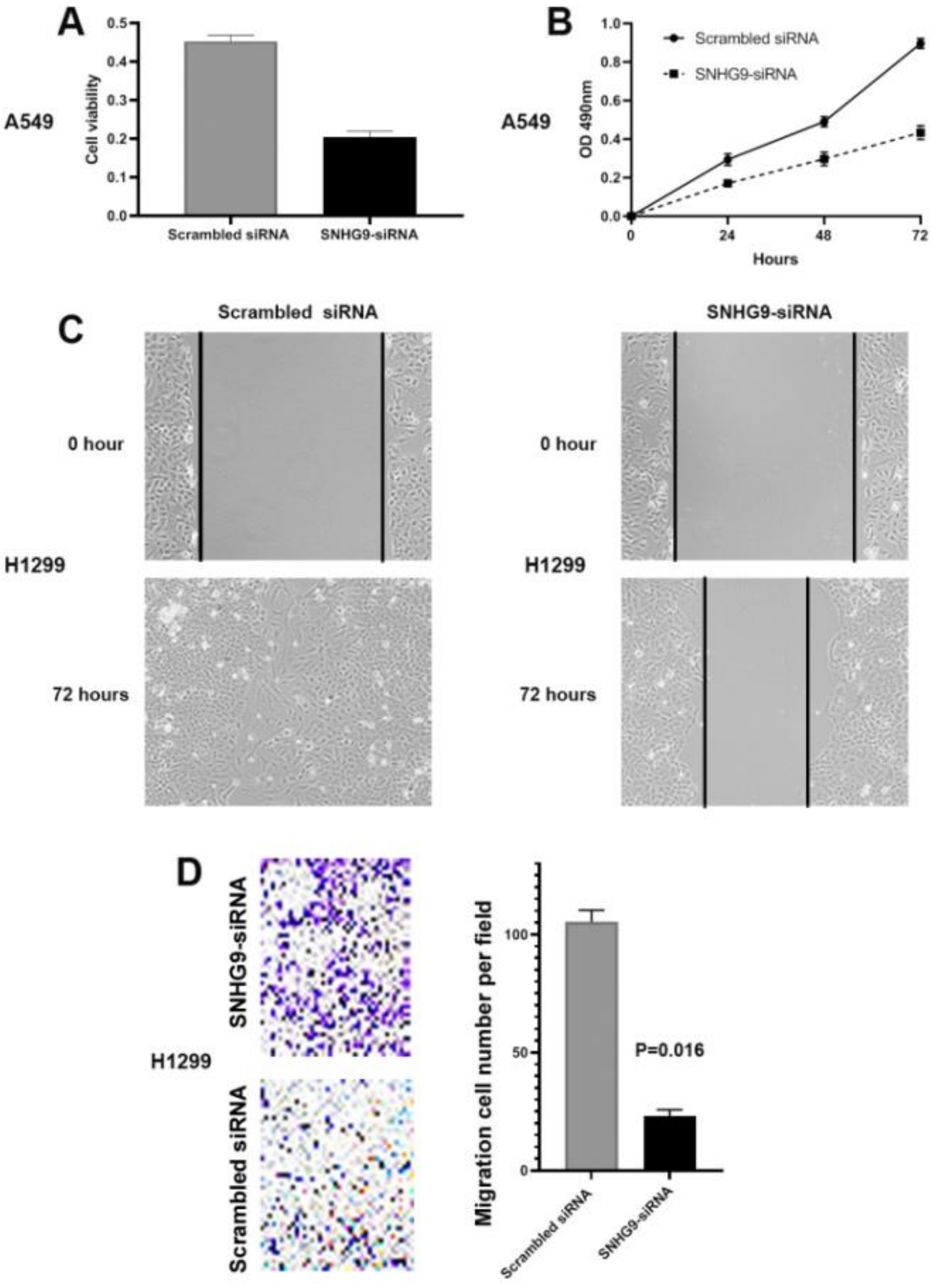

Supplementary Figure 4. SNHG9 knockdown inhibits the tumorigenicity of lung cancer cells. (A). SNHG9 knockdown can significantly reduce A549 cell viability 72 hours. The cell viability is determined by a cell viability assay using Cell Counting Kit 8 (Abcam). (B). SNHG9 knockdown can suppress cell proliferation in the A549 cancer cells. (C). In the wound-healing assays, H1299 cells transfected with SNHG9-siRNA show a slower gap closure compared with cells transfected with scrambled siRNA. (D), transwell migration assays show that SNHG9 knockdown can constrain migration and invasion of H1299 cancer cells. The migratory cells are counted and the results are expressed as the mean number of migratory cells ± SD/selected microscopic field (n = 5). The figure shows the results from the time point 24 hours of H1299 cells.

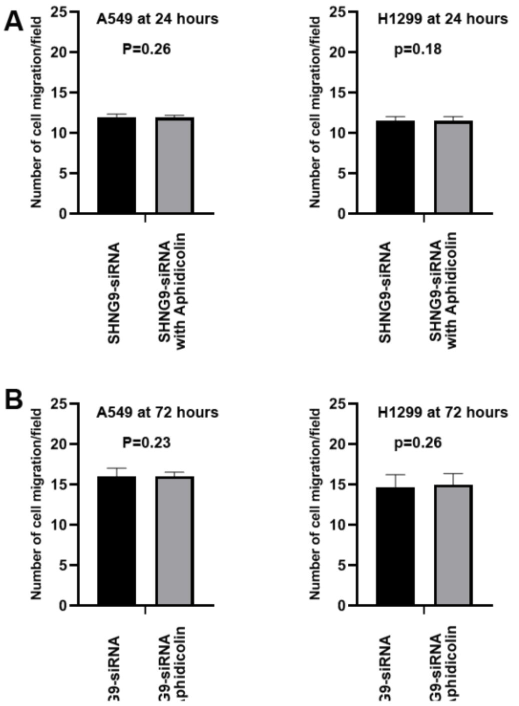

Supplementary Figure 5. Effect of SNHG9 knockdown on cell migration is not associated with the presence of a proliferation inhibitor. To determine if the inhibition of cell migration by SNHG9 knockdown is due to the inhibition of the cell proliferation, cancer cells transfected with siRNA-SNHG9 are treated with aphidicolin (1 mg/mL), a proliferation inhibitor, for 24 h (A) or 72 h (B). Aphidicolin does not affect cell migration state of both A549 and H1299 cancers treated with SNHG9 knockdown (all p>0.05), suggesting that the phenotype is not due to the observed differences in proliferation.

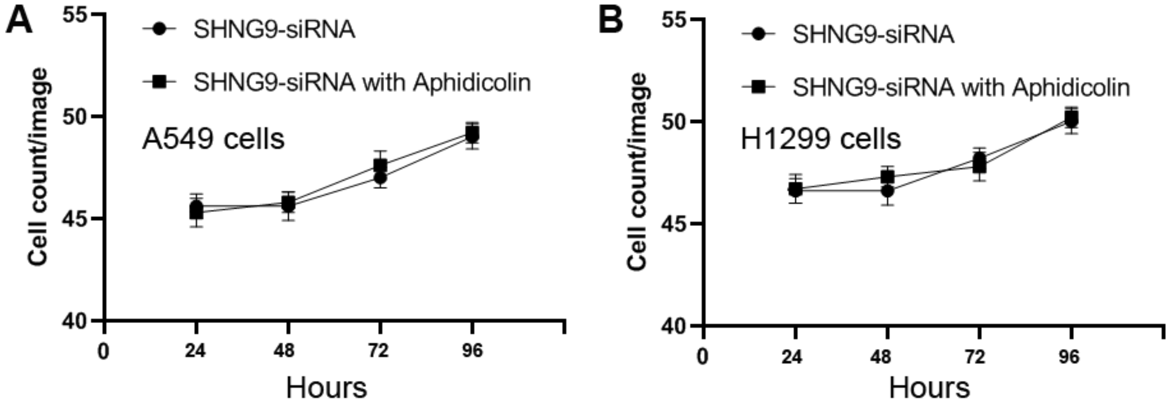

Supplementary Figure 6. Effect of SNHG9 knockdown on cell proliferation is not associated with the presence of a proliferation inhibitor. Cell counts were measured every 24 hour for 96 hours. A continuous cell count was measured by a cell imager in cells/image. All experiments represent the mean of three independent experiments, ± the S.D. Aphidicolin does not affect cell migration state of A549 (A) and H1299 (B) cancers treated with SNHG9 knockdown (all p>0.05), suggesting that the phenotype is not due to the observed differences in proliferation.

© 2019 by the authors. Submitted for possible open access publication under the terms and conditions of the Creative Commons Attribution (CC BY) license (http://creativecommons.org/licenses/by/4.0/).
